# Faecal haemoglobin concentrations in women and men diagnosed with colorectal cancer in a national screening programme

**DOI:** 10.1177/09691413211056970

**Published:** 2021-11-22

**Authors:** Gavin RC Clark, Jayne Digby, Callum G Fraser, Judith A Strachan, Robert JC Steele

**Affiliations:** 1 9571 Public Health Scotland, Edinburgh, Scotland, UK; 2 Centre for Research into Cancer Prevention and Screening, 85326 University of Dundee, Dundee, Scotland, UK; 3 Blood Sciences and Scottish Bowel Screening Laboratory, Dundee, Scotland, UK

**Keywords:** Colorectal cancer screening, colorectal cancer site, colorectal cancer stage, faecal immunochemical test, faecal haemoglobin, gender differences

## Abstract

**Objective:**

There is evidence that colorectal cancer screening using faecal haemoglobin is less effective in women than men. The faecal haemoglobin concentrations were therefore examined in women and men with screen-detected colorectal cancer.

**Setting:**

Scottish Bowel Screening Programme, following the introduction of a faecal immunochemical test from November 2017, to March 2020**.**

**Methods:**

Data were collated on faecal haemoglobin concentrations, pathological stage and anatomical site of the main lesion in participants who had colorectal cancer detected. The data in women and men were compared.

**Results:**

For the faecal haemoglobin concentrations studied (>80 µg Hb/g faeces), the distributions indicated lower concentrations in women. Marked differences were found between women and men diagnosed with colorectal cancer. The median faecal haemoglobin concentration for women (*n* = 720) was 408 µg Hb/g faeces compared to 473 µg Hb/g faeces for men (*n* = 959) (*p* = 0.004) and 50.6% of the results were >400 µg Hb/g faeces in women; in men, this was 57.8%. The difference in faecal haemoglobin concentrations in women and men became less statistically significant as stage advanced from stages I–IV. For right-sided, left-sided and rectal colorectal cancer, a similar gender difference persisted in all sites. Differences in faecal haemoglobin between the genders were significant for left-sided cancers and stage I and approached significance for rectal cancers and stage II, but all sites and stages showed lower median faecal haemoglobin concentrations for women.

**Conclusions:**

To minimise gender inequalities, faecal immunochemical test-based colorectal cancer screening programmes should evaluate a strategy of using different faecal haemoglobin concentration thresholds in women and men.

## Introduction

Screening for colorectal cancer (CRC) using the detection of faecal haemoglobin (f-Hb) creates gender inequality. Although women are consistently more likely to participate in faecal test-based screening strategies than men, the yield of neoplastic pathology is lower in women.^1–3^ For example, in the first year of the Scottish Bowel Screening Programme (SBoSP) after the introduction of a faecal immunochemical test (FIT) to determine the f-Hb concentration, 0.09% of all women (*n* = 307,919) and 0.15% of all men (*n* = 279,530) screened were diagnosed as having invasive CRC, and 0.36% of women versus 0.80% of men had higher risk adenomas.^2^ These findings may be explained partially by the lower incidence of CRC in women,^4^ and the tendency for CRC in women to be right-sided and, consequently, less amenable to detection using f-Hb.^5^ However, there is now consistent evidence that women are more likely to present with interval CRC (i.e. CRC diagnosed within two years of a “negative” screening test result),^6,7^ indicating that screening using f-Hb is less sensitive for CRC in women than in men. In addition, there is strong evidence from trials of CRC screening based on f-Hb that the CRC mortality reduction gained is less in women than in men.^8,9^

To date, this evidence is largely based on the guaiac faecal occult blood test (gFOBT). However, these tests are considered obsolete^10^ and screening programmes world wide,^11^ including all in the United Kingdom, and many in Europe,^12^ have replaced, or are replacing, the gFOBT with the FIT. Unlike the gFOBT, the FIT is specific for human haemoglobin (Hb) and can provide a quantitative estimate of the f-Hb concentration. It is known that in screening populations, and probably in the population at large, f-Hb concentrations are lower in women than in men,^13,14^ so that, for any given screening f-Hb concentration threshold, the positivity in women is lower than that in men. For example, in the first year of the FIT-based programme in Scotland, which uses a threshold of >80 µg of Hb per gram of faeces (µg Hb/g faeces), the positivity was 2.6% in women and 3.6% in men, respectively.^2^

However, this in itself does not explain the poorer CRC detection rates and higher interval cancer rates in women, and it would seem likely that the f-Hb concentrations in women with CRC would be lower than in men with comparable lesions. If this is the case, it would lend support to the view that f-Hb concentration thresholds for screening should be lower in women than in men, but, to our knowledge, this has not been studied to date.

We have therefore interrogated the data from the FIT-based SBoSP to determine the f-Hb concentrations in women and men detected with CRC, taking into account the anatomical location of the main lesion within the large bowel and the pathological stage.

## Methods

### Data analysis

As previously described,^2^ the SBoSP began with three pilot screening rounds in three of the 14 National Health Service Boards in Scotland. The national roll-out of the SBoSP using a two-tier reflex gFOBT/qualitative FIT algorithm then began in 2007 and was complete by the end of 2009. This strategy was used until 2017 when FIT as a first-line test was introduced. Data from the SBoSP between the introduction of FIT in November 2017 and March 2020 were used for this study. Of those who had “positive” test results (i.e. with an f-Hb concentration >80 µg Hb/g faeces), the f-Hb concentrations in women (17,732) were compared with those in men (22,857). As the time period covers more than a single two-year screening round, there were a small number of participants with two positive test results; in this instance the first f-Hb concentration result was used to ensure homogeneity of the cohort studied. The same was done for those diagnosed with CRC and for those diagnosed with CRC at different stages and in different sites within the large bowel. The cancer stage categories used were I–IV (I – T1-2, N0, M0, Dukes’ Stage A; II – T3-4, N0, M0, Dukes’ Stage B; III – T1-4, N1-2, M0, Dukes’ Stage C; IV – T1-4, N0-2, M1, Dukes’ Stage D). Cancer anatomical site was classified using ICD10 codes^15^: codes C18-18.5 were classified as right-sided cancers, C18.6-18.7 were classified as left-sided, C19-20 were classified as rectal, and those cancers with ICD10 codes C18.8-18.9 or where no ICD10 code was available were classified as site unknown.

### F-Hb concentration determination

The logistics involved in the FIT-based SBoSP have been described in detail previously.^2^ In brief, participants in the SBoSP returned the completed FIT specimen collection device by post to the Scottish Bowel Screening Laboratory (Ninewells Hospital and Medical School, Dundee). Analyses were carried out from Monday to Friday; most devices were analysed on the day of receipt and the f-Hb results were reported electronically to the Scottish Bowel Screening System (BoSS) after f-Hb concentration measurement using one of four HM-JACKarc (Hitachi Chemical Diagnostics Systems, Tokyo, Japan) FIT systems. The Laboratory has ISO (International Organization for Standardization) 15,189 accreditation. Total quality management is comprehensively practised, including internal quality control and external quality assessment carried out by the UK National External Quality Assessment Scheme (UKNEQAS). Use of data outside the analytical measurement range documented by the manufacturer, which is 7–400 µg Hb/g faeces for the FIT system used in this study, has become usual for research as well as clinical purposes at low f-Hb concentrations below the limit of detection, and this approach has been supported in the peer-reviewed literature.^16^ We have adopted an analogous strategy through examination of all results >80 µg Hb/g faeces, including those greater than the upper measurement limit of 400 µg Hb/g faeces.

### Statistical analyses

Statistical significance for the difference in f-Hb concentrations between women and men was assessed using the Wilcoxon Rank Sum test; *p*-values were considered significant at the 0.05 level. R statistical software v3.6.1 was used for all data processing and analysis.^17^

## Results

**Figure 1.**
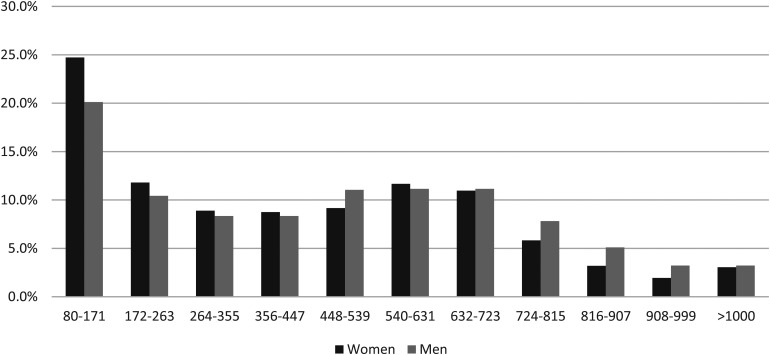
Percentages of women and men with screen-detected colorectal cancer by faecal haemoglobin concentration class (µg Hb/g faeces).

**Figure 2.**
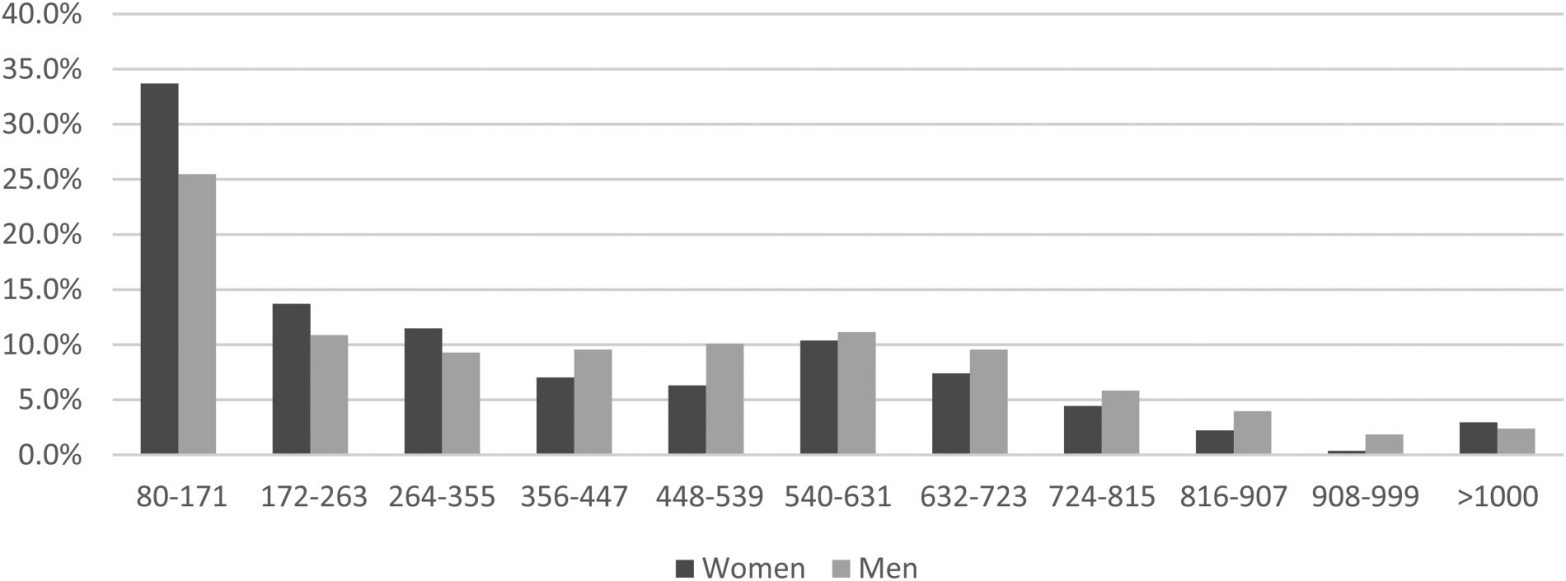
Percentages of women and men with stage I screen-detected colorectal cancer by faecal haemoglobin concentration class (µg Hb/g faeces).

Table 1 shows the median f-Hb concentrations, percentages of results with f-Hb concentration >400 µg Hb/g faeces, and *p*-values (Wilcoxon Rank Sum Test), for women and men with screen-detected CRC, in total and subdivided by stage and site. For the f-Hb concentrations studied (>80 µg Hb/g faeces), a marked difference was found between women and men, of all those diagnosed with CRC. The distributions of f-Hb concentrations in all those women and men with CRC and those with stage I disease are shown in Figures 1 and 2. Similar figures for the f-Hb distributions in stages II–IV and for right-sided, left-sided, and rectal CRC are provided in the Supplementary Figures.

**Table 1.** Median faecal haemoglobin concentration (faecal haemoglobin (f-Hb), µg Hb/g faeces), percentage with faecal haemoglobin concentration >400 µg Hb/g faeces and *p*-value comparing women and men by gender, stage and site for participants with colorectal cancer.

	Gender	*N*	Median f-Hb (µg Hb/g faeces)	Percentage with f-Hb >400 µg Hb/g faeces	*p*-value
Total	Women	720	408	50.5	0.004
	Men	959	473	57.8	
Stage I	Women	270	284	38.5	0.008
	Men	377	396	49.1	
Stage II	Women	174	510	59.2	0.055
	Men	191	551	69.6	
Stage III	Women	180	465	58.3	0.232
	Men	256	512	60.9	
Stage IV	Women	51	549	64.7	0.836
	Men	69	582	66.7	
Stage unknown	Women	43			
	Men	68			
Right-sided	Women	300	422	52.0	0.3851
	Men	272	482	59.6	
Left-sided	Women	244	406	50.4	0.0145
	Men	331	484	59.8	
Rectal	Women	175	389	48.0	0.0502
	Men	351	471	55.0	
Site unspecified	Women	1			
	Men	5			

The median f-Hb concentration for all screen-detected CRC women was 408 µg Hb/g faeces compared to 473 µg Hb/g faeces for men (*p* = 0.004) and 50.6% of the results were >400 µg Hb/g faeces in women whereas in men this was 57.8%. Differences in f-Hb between the genders were statistically significant for left-sided cancers and stage I and approached significance for rectal cancers and stage II. All stages and sites showed lower median f-Hb concentrations for women, but it should be noted that the difference between women and men decreased with increasing stage. In 111 cases, stage information was not available.

## Discussion

Since women do have lower f-Hb concentrations than men,^13,14^ it is not surprising that, in screening programmes based on the use of a single f-Hb concentration threshold, positivity is found to be lower in women than in men. What is less easy to explain is the lower CRC detection rate, the higher interval cancer rate, and the smaller effect of screening on CRC-specific mortality, since it might be expected that CRC would bleed to the same extent in women and men. Thus, the explanations of a lower CRC incidence and more right-sided lesions in women than men are attractive, although a previous analysis of a FIT-based screening pilot in Scotland has indicated that interval cancers were more common in women irrespective of anatomical site.^5^

This issue is clarified by the findings presented here which indicate that stage for stage and site for the site within the large bowel, CRCs are associated with lower f-Hb concentrations in women than in men. This could be explained by the lower background f-Hb concentration in women, such that a CRC would have to bleed more in a woman than a man to render the f-Hb concentration above the pre-determined threshold that triggers a further investigation, usually a colonoscopy. It is interesting that the difference between women and men becomes less with more advanced CRC. This may be related to sample size, since early CRCs are more common in a screening programme than the later disease as evidenced by the data presented here. However, an alternative explanation might be that because more advanced CRCs bleed more than early cancers, the effect of differential background f-Hb concentrations is reduced. Given that the mortality advantage of screening is dependent on the detection of early disease, the large difference between women and men in early disease that we have observed is of particular significance.

Why women have lower f-Hb concentrations than men is by no means clear. Blood Hb concentrations tend to be lower in women than men, and it has been suggested that f-Hb concentrations could mirror this; however, this seems implausible given the age range invited for screening (50–74 years) and that the small difference disappears a few years after the menopause.^18^ Another possibility is related to the intrinsically lower bowel transit times in women,^19^ and it is, therefore, possible that degradation of f-Hb before defaecation occurs to a greater extent in women than in men. Similarly, more degradation of any f-Hb present in women as compared to men could be due to slower transit caused by constipation, much common in women than men.^20^ An alternative explanation is presented by the observation that f-Hb is related to death from multiple causes unrelated to CRC^21^ and to the prescription of medicines that do not cause gastrointestinal bleeding for a range of conditions including heart disease, hypertension, depression, and type two diabetes.^22^ Since these conditions all have an inflammatory component, it has been hypothesised that f-Hb is an index of systemic inflammation^21,22^ but, whatever the reason, f-Hb is a marker for poor health. Thus, an intriguing possibility is that, in participants who have detectable f-Hb but no significant disease on colorectal visualisation, such f-Hb identifies those who are at a high risk of harbouring or developing chronic conditions.^22^ In Scotland, it is well established that women have a healthier lifestyle than men and they are also less likely to suffer from chronic non-communicable diseases.^23^ Thus, the lower f-Hb in women may reflect less systemic inflammation in women than in men.

Whatever the explanation for the findings presented in this communication, employing a lower f-Hb concentration threshold for women than for men should alleviate the current gender inequality, since this is highly likely to bring the CRC detection and interval cancer rates in women more in line with those in men. This would, of course, generate an increased number of colonoscopies for women and there is also a concern that, since the positive predictive value (PPV) falls with decreasing f-Hb concentration thresholds,^24^ and since the PPV is lower in women than in men in gFOBT screening,^6^ there might be a significant burden of “false positive” test results in women. However, recent Scottish data indicated that using FIT at an f-Hb concentration threshold of >80 µg Hb/g faeces, the PPV for CRC in women was 5.0%, similar to that in men (5.3%).^2^ In addition, a recent study from Finland showed that using >25 μg Hb/g faeces for women and >70 μg Hg/g faeces for men gave similar CRC detection rates in both genders (0.16% for women and 0.18% for men) and similar PPV for CRC (6.4% for women and 6.6% for men).^25^ However, in this Finnish study, the positivities in women and men using these thresholds were 2.6% and 2.4%, respectively. These positivities are considerably lower than those currently observed at similar thresholds in Scotland, perhaps due to demographic factors such as lifestyle, and also because f-Hb concentrations found are highly dependent on the FIT system employed,^26^ so the results from Finland are not necessarily transferable over geography. A recent study in Sweden investigated participants in screening using f-Hb concentration thresholds of >40 µg Hb/g faeces in women and >80 µg Hb/g faeces in men.^27^ The yield of CRC was assessed and compared to a threshold of >80 µg Hb/g faeces in both genders. Positivity in this Swedish study was 2.7% in both genders but in this case, the PPV for CRC was significantly lower in women (5.8%) than men (8.3%). “Negative” colonoscopies were, therefore, more common in women (24%) than in men (17%), but in 120 women with CRC, 28 (23.3%) had f-Hb concentrations ≤80 µg/g. Thus, it was concluded that the high rate of CRC detected in women using the lower f-Hb threshold outweighed the minor increase in screening costs incurred by using gender-sensitive differential thresholds.

CRC incidences vary from one country to another; f-Hb distributions also vary between countries^28^ and even between regions of a small country like Scotland.^14^ In addition, in FIT-based screening programmes, different f-Hb concentration thresholds are employed to take account of national colonoscopy capacities.^11,12^ Thus, the issue of whether or not partitioned f-Hb thresholds should be used for women and men can only be resolved by prospective, observational studies. The distributions of f-Hb concentrations in men and women with CRC, the newer data from Scandinavia,^25,28^ and other evidence on screening outcomes, as detailed above, indicate that lowering the f-Hb concentration threshold in women would increase the chances of detecting CRC in those participating in screening. However, there are areas of significant uncertainty and these include the extent to which CRC detection rates in women and men would converge, the effect on the PPV and the consequent harms of any increase in the false positive rate, the effect on interval cancer rates in women and the cost-effectiveness of this strategy.

Given the quantitative nature of the FIT used in the SBoSP and in most other programmatic CRC screening, it would be straightforward to set the screening threshold at different f-Hb concentrations for women and men. Data from the SBoSP indicates that a threshold of >50 µg Hb/g faeces would yield a positivity of 3.7% in women in Scotland, almost identical to that in men at the current threshold of >80 µg Hb/g faeces used for both genders. In order to test the hypothesis that lowering the f-Hb concentration threshold in women to achieve the same positivity as that seen in men will improve screening outcomes for women in Scotland, a simple cohort study in which a consecutive series of women invitees are offered a FIT which is reported as warranting further investigation at a threshold of >50 µg Hb/g faeces would likely suffice. An important consideration in any research performed in a fully rolled out successful national screening programme is that no participant is disadvantaged. In this case, the parameters used in the SBoSP applicable to men would remain unchanged and it is postulated that more women would be advantaged.

## Conclusions

To obviate the clear gender inequalities occasioned by using the same f-Hb concentration threshold for women and men, we urge all countries with national or regional FIT-based CRC screening programmes to evaluate a strategy of using different f-Hb concentration thresholds in women and men such that gender-related differences in positivity are eliminated. This would provide the evidence required to decide whether or not a gender-based approach is effective in a country- or region-specific CRC screening programme. This simple approach could be a first step to the initiation of stratified approaches to CRC screening, such as those recently advocated which involve additional variables.^29^

## Supplemental Material

sj-docx-1-msc-10.1177_09691413211056970 - Supplemental material for Faecal haemoglobin concentrations in women and men diagnosed with colorectal cancer in a national screening programme Click here for additional data file. Supplemental material, sj-docx-1-msc-10.1177_09691413211056970 for Faecal haemoglobin concentrations in women and men diagnosed with colorectal cancer in a national screening programme by Gavin RC Clark, Jayne Digby, Callum G Fraser, Judith A Strachan and Robert JC Steele in Journal of Medical Screening
