# Supplementary material for: Faecal haemoglobin concentrations in women and men diagnosed with colorectal cancer in a national screening programme
Source: J Med Screen. 2021 Nov 22;29(1):26–31. doi: 10.1177/09691413211056970 (PMC8892068; doi:10.1177/09691413211056970)
Supplement: sj-docx-1-msc-10.1177_09691413211056970 - Supplemental material for Faecal haemoglobin concentrations in women and men diagnosed with colorectal cancer in a national screening programme [file sj-docx-1-msc-10.1177_09691413211056970.docx]

**Supplementary Figures**

**Percentages of women and men with Stage II–IV (Figures S1.1–S1.3) screen-detected colorectal cancer, and grouped by site (Figures S1.4–S1.6), by faecal haemoglobin concentration class (µg Hb/g faeces).**

**Figure S1.1. Percentage of women and men with Stage II screen-detected colorectal cancer by faecal haemoglobin concentration class (µg Hb/g faeces).**

|  | \|  \| \| --- \| |  |  |  |  |  |  |  |  |  |  |  |  |  | **Wilcoxon test for significance** | |
| --- | --- | --- | --- | --- | --- | --- | --- | --- | --- | --- | --- | --- | --- | --- | --- | --- | --- |
|  |  |  |  |  |  |  |  |  |  |  |  |  |  |  | p | 0.00 |
| **Figure S1.2. Percentage of women and men with Stage III screen-detected colorectal cancer by faecal haemoglobin concentration class (µg Hb/g faeces).** |  |  |  |  |  |  |  |  |  |  |  |  |  |  |  |  |
|  |  |  |  |  |  |  |  |  |  |  |  |  |  |  |  |  |
|  |  |  |  |  |  |  |  |  |  |  |  |  |  |  |  |  |
|  |  |  |  |  |  |  |  |  |  |  |  |  |  |  |  |  |
|  |  |  |  |  |  |  |  |  |  |  |  |  |  |  |  |  |
|  |  |  |  |  |  |  |  |  |  |  |  |  |  |  |  |  |
|  |  |  |  |  |  |  |  |  |  |  |  |  |  |  |  |  |
| **Figure S1.3. Percentage of women and men with Stage IV screen-detected colorectal cancer by faecal haemoglobin concentration class (µg Hb/g faeces).** |  |  |  |  |  |  |  |  |  |  |  |  |  |  |  |  |
|  |  |  |  |  |  |  |  |  |  |  |  |  |  |  |  |  |
|  |  |  |  |  |  |  |  |  |  |  |  |  |  |  |  |  |
| **Figure S1.4. Percentage of women and men with right-sided screen-detected colorectal cancer by faecal haemoglobin concentration class (µg Hb/g faeces).** |  |  |  |  |  |  |  |  |  |  |  |  |  |  |  |  |
|  |  |  |  |  |  |  |  |  |  |  |  |  |  |  |  |  |
|  |  |  |  |  |  |  |  |  |  |  |  |  |  |  |  |  |
|  |  |  |  |  |  |  |  |  |  |  |  |  |  |  |  |  |
|  |  |  |  |  |  |  |  |  |  |  |  |  |  |  |  |  |

| **Figure S1.5. Percentage of women and men with left-sided screen-detected colorectal cancer by faecal haemoglobin concentration class (µg Hb/g faeces).** |  |  |  |  |  |  |  |  |  |  |  |  |  |  |  |  |
| --- | --- | --- | --- | --- | --- | --- | --- | --- | --- | --- | --- | --- | --- | --- | --- | --- |
|  |  |  |  |  |  |  |  |  |  |  |  |  |  |  |  |  |
|  |  |  |  |  |  |  |  |  |  |  |  |  |  |  |  |  |

**Figure S1.6. Percentage of women and men with rectal screen-detected colorectal cancer by faecal haemoglobin concentration class (µg Hb/g faeces)**
